# Comparative evaluation of liver, spleen, and kidney stiffness in HIV-monoinfected pediatric patients via shear wave elastography

**DOI:** 10.3906/sag-1811-87

**Published:** 2019-06-18

**Authors:** Emine ÇALIŞKAN, Gürkan ATAY, Manolya KARA, Murat SÜTÇÜ, Zuhal BAYRAMOĞLU, Selda HANÇERLİ TÖRÜN, Ayper SOMER, İbrahim ADALETLİ

**Affiliations:** 1 Department of Pediatric Radiology, Seyhan State Hospital, Adana Turkey; 2 Department of Pediatrics, Faculty of Medicine, İstanbul University, İstanbul Turkey; 3 Department of Pediatric Infectious Diseases, Faculty of Medicine, İstanbul University, İstanbul Turkey; 4 Department of Pediatric Infectious Diseases, Konya Training and Research Hospital, Konya Turkey; 5 Department of Pediatric Radiology, Faculty of Medicine, İstanbul University, İstanbul Turkey

**Keywords:** Children, HIV, shear wave elastography, stiffness

## Abstract

**Background/aim:**

This study aimed to evaluate the stiffness of the liver, spleen, and kidneys in HIV-monoinfected children via shear wave elastography (SWE).

**Materials and methods:**

Twenty-one HIV-monoinfected children and 37 healthy subjects were included in this study. Livers, spleens, and kidneys of the participants were examined via ultrasound and SWE. Patients were divided into two groups according to the presence of pathologic ultrasonographic findings. Routine laboratory tests were also recorded. Stiffness of these organs was compared between patients and control groups.

**Results:**

Liver transaminases, blood urea, and creatinine levels were normal in all subjects. Ultrasonographic examination revealed hepatosplenomegaly (n = 1, 4.7%), grade 1 hepatosteatosis (n = 1, 4.7%), and hepatosteatosis and minimal heterogeneity of the liver (n = 1, 4.7%). Ultrasonographic features were normal in 18 patients. Stiffness of the liver, spleen, and kidneys of all HIV-monoinfected children with normal laboratory parameters was significantly higher than in healthy subjects. Eighteen patients with normal ultrasonographic findings also had higher stiffness values when compared to control subjects.

**Conclusion:**

Stiffness of the liver, spleen, and kidneys in HIV-monoinfected children was increased. SWE can be used in the detection of early parenchymal changes even in patients with normal laboratory parameters and ultrasonographic findings.

## 1. Introduction

Liver involvement can cause mortality and morbidity in patients with human immunodeficiency virus (HIV) infection. Although coinfection with hepatitis B and C viruses remains the major factor causing liver damage, long-term use of antiretroviral therapy (ART), cytotoxic effects, and prolonged inflammation stimulated by HIV together with metabolic complications also contribute to liver fibrosis [1,2]. Similarly, portal hypertension secondary to chronic liver disease, immunologic activation, and malignancy can change the parenchymal architecture of the spleen [3,4]. Glomerular and tubular interstitial involvements can also cause acute and chronic renal disorders in HIV-infected children [5,6].

Ultrasound elastography is a radiologic technique used to measure tissue stiffness. Tissue stiffness measurements may be beneficial for the identification and treatment of a variety of organ disorders [7–11]. Strain elastography and acoustic radiation force impulse elastography were the first methods developed for this purpose [12,13]. However, the overall use of shear wave elastography (SWE) has increased in recent years, since it is easy to apply, is less radiologist-dependent, and gives quantitative results [12–14]. 

Owing to highly developed ART, HIV infection is now a chronic disorder with long life expectancy. On the other hand, HIV-related cytopathies are now major concerns of physicians [15]. Earlier radiologic detection of these toxic effects via SWE can aid in determining organ damage and be useful for the early diagnosis and management of the disease. This approach can extend the life expectancy of HIV patients, particularly children who are exposed to the virus and ART for a longer period. As far as we know, there is no study evaluating the organ stiffness of HIV-monoinfected children by SWE. 

In this study, we evaluated the stiffness of the liver, spleen, and both kidneys in HIV-monoinfected children via SWE in order to determine the applicability of this method for early detection of parenchymal damage.

## 2. Materials and methods

This was a single-center, observational cross-sectional study performed between September 2017 and December 2017. The study included 21 HIV-monoinfected children (age interval: 0.5–18.5 years) followed in our Pediatric Infectious Diseases Department and 37 healthy volunteer children (age interval: 0.5–18 years). The liver, spleen, and both kidneys of all participants were examined via ultrasonography (US) and SWE. Laboratory test results were routinely performed and recorded during patient follow-up including complete blood count, liver transaminases, blood urea nitrogen, creatinine levels, gamma-glutamyl transferase (GGT), alkaline phosphatase (ALP), bilirubin, albumin, amylase, triglycerides, low-density lipoprotein (LDL), high-density lipoprotein (HDL), HIV RNA copy levels, CD4+ T cell count, and urine analysis. 

### 2.1. Patients and control group

The same method was applied to the patients and the control group and they were evaluated consecutively. The HIV-infected patients, either congenitally or acquired, were referred to the radiologists by the pediatric infectious disease specialists during routine follow-up visits. The control group included healthy subjects who presented to the radiology outpatient clinic for other reasons with no chronic disease and no chronic drug use and who had normal US findings. The patient and control groups were age- and sex-matched. All patients were evaluated radiologically once. The ones with adequate radiologic evaluation were included in the study. The radiologists were not blinded to the HIV diagnosis of the patients. Power analysis was used to determine the number of children in the control group. Type 1 error was calculated as 0.05, type 2 error was 0.20, and the effect size was at least 30 in the control group. 

Among 24 children with HIV infection, 2 were excluded because of HBV and HCV coinfection and one refused to participate in the study. The patient group thus contained 21 HIV-monoinfected children.

Forty-one healthy children with no known systemic illnesses volunteered to participate in the study. Among those, one child with splenomegaly, one child with fatty liver, and 2 children who were unable to cooperate during SWE examination were excluded. Finally, 37 healthy subjects with no clinical symptoms and normal US findings of the mentioned organs were included in the control group.

Body weight, height, and body mass index (BMI) of all children were measured before SWE examination. Stiffness values of the liver, spleen, and both kidneys of HIV-monoinfected children and healthy subjects were compared.

Demographic, clinical, and laboratory characteristics of HIV-monoinfected children regarding the duration of illnesses, ART, HIV RNA copy levels, and blood chemistry results were recorded. Patients were subgrouped according to viral load (>45 copies/mL and undetectable) and the duration of illness (more or less than 5 years). Stiffness of the mentioned organs was also compared within patient subgroups.

### 2.2. SWE technique

Measurements were examined using an Aplio 500 Platinum ultrasound device (Canon Medical Systems, Japan) with a convex transducer (frequency range: 1.9–6 MHz) for the liver, spleen, and both kidneys. SWE measurements were completed with the common consensus of two experienced pediatric radiologists with more than 5 years of elastography experience. A sufficient amount of ultrasound gel was used during elasticity measurements. While obtaining the SWE images, no pressure was applied to the probe and it was ensured that the operator’s hand was steady.

**Figure 1:**
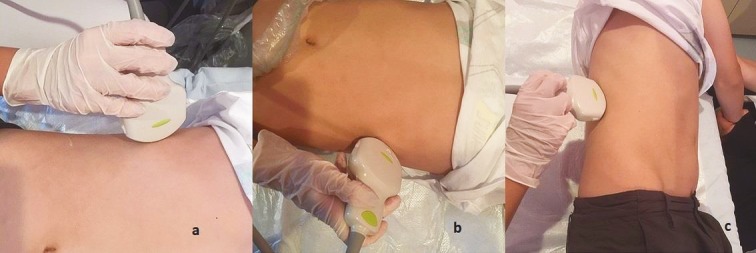
Patient positions for SWE examination. Liver and spleen were examined using intercostal approach in supine position (a and b). Lateral decubitus position was used for kidneys (c).

The SWE technique was applied in supine position for the liver and spleen and in lateral decubitus position for the kidneys. The liver and spleen were examined via an intercostal approach (Figures 1a and 1b). The kidneys were examined in the lumbar region of the back (Figure 1c). The 2D-SWE map and quality mode were examined in split-screen mode.

**Figure 2:**
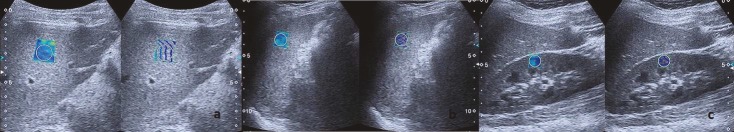
Images of SWE measurements. Liver (a) and spleen (b) examinations were completed using a circular ROI that was placed on the parenchyma. ROI was placed on cortex for kidneys (c). 2D-SWE map (left sides) and quality mode (right sides) are seen.

The quality mode, which is identified as the propagation mode (arrival time contour), is a mode in which reliable data are obtained when the lines are parallel and smooth, and the increase in distance between lines is parallel to the increase in stiffness. Subsequently, a region of interest (ROI) of 5 mm in diameter was placed to the parenchyma that did not contain vascular structures for the liver, spleen, and kidneys, and five repeated acquisitions were obtained. SWE measurements were performed 2 cm below the capsule of the liver and spleen, and through the renal cortex for the kidneys (Figures 2a–2c). The median value of the measured data points was calculated. The elasticity modulus (kPa: range 0–80) and shear wave velocity modulus (m/s: range 0–8) were used for measurements.

### 2.3. Statistical analysis

Statistical analysis of data was performed with SPSS 21.0 for Windows (IBM Corp., Armonk, NY, USA). Normality was tested using the Shapiro–Wilk and Kolmogorov–Smirnov tests. Age, BMI, and stiffness of the liver, spleen, and kidneys were given as median, minimum, and maximum or mean ± standard deviation. The continuous variables between HIV-monoinfected children and the control group and between subgroups of HIV-monoinfected children were compared using the Mann–Whitney U test. Variables were investigated at the 95% confidence interval with P < 0.05 accepted as significant.

## 3. Results

The mean ages of 21 HIV-monoinfected children and the control group (n = 37) were 11.1 ± 4.4 years and 11.6 ± 4.9 years, respectively. The mean BMI was 17.2 ± 5.7 for HIV patients and 18.0 ± 5.2 for the control group. The patient group had 11 male children (52.3%) and the control group had 19 (51.3%). There was no statistical difference between patient and control groups in terms of age, sex, and BMI. The median duration of the illness in the patient group was 6.0 (0.5–16.3) years. 

Detailed patient data are summarized in Tables 1 and 2.

**Table 1:** Data of stiffness values in HIV-monoinfected children.

n	Liver SWE		Spleen SWE		Right kidney SWE		Left kidney SWE	
	kPa	m/s	kPa	m/s	kPa	m/s	kPa	m/s
1	9.9	1.7	17.3	2.1	21.4	2.6	21.8	2.6
2	25.5	2.9	Splenectomy	Splenectomy	15.8	2.2	15.3	2.2
3	9.1	1.7	28.9	3.2	15.1	2.2	15.8	2.3
4	7.2	1.5	18.4	2.4	18.6	2.5	18.3	2.4
5	9.2	1.7	24.2	2.8	17.3	2.4	17.5	2.4
6	7.4	1.5	16.2	2.3	17.5	2.3	18.3	2.4
7	8.5	2	18.9	2.5	16.2	2.3	15.8	2.2
8	11.4	1.8	18.5	2.4	18.4	2.4	18.3	2.4
9	8.8	1.7	21.4	2.6	20.9	2.6	20.2	2.5
10	8.9	1.7	15.1	2.2	20.5	2.5	17.8	2.4
11	7.1	1.6	24.1	2.5	17.8	2.4	18.2	2.4
12	6.6	1.4	18.1	2.4	18.6	2.4	18.1	2.4
13	6.6	1.4	15.7	2.2	18.5	2.4	18.2	2.4
14	7.2	1.6	18.6	2.4	22.9	2.6	10.1	1.8
15	8.7	1.7	17.9	2.4	19.5	2.5	20.2	2.6
16	8.6	1.7	22.1	2.7	15.9	2.2	16.1	2.3
17	9.9	1.5	26.3	2.5	19.5	2.2	19.1	2.3
18	6.7	1.5	20.1	2.3	13.8	2.1	13.5	2.1
19	4.4	1.2	15.7	2.2	12.9	2.1	12.8	2.0
20	8.4	1.6	19.3	2.5	18.3	2.4	18.4	2.5
21	10.1	1.8	27.5	2.9	19.8	2.3	19.8	2.3

SWE: Shear wave elastography.

**Table 2:** Age, sex (M/F), duration of illnesses (d), HIV RNA values, and antiretroviral treatments of HIV infected children.

n	Age (years)	M/F	d(years)	HIV RNAcopies/mL	ART
1	15.5	F	7.0	UD	3TC, AZT, LPV/r
2	16.5	M	16.3	UD	3TC, AZT, LPV/r
3	18.5	F	9.0	UD	3TC, AZT, LPV/r
4	8.3	M	5.0	478,000	AZT, LPV/r, TDF
5	12.4	M	4.0	UD	EFV, 3TC, DTG
6	10.0	F	9.9	44,080	AZT, LPV/r, TDF
7	11.8	F	11.0	UD	3TC, AZT, LPV/r
8	14.7	F	3.0	UD	3TC, AZT, LPV/r
9	9.4	F	9.4	UD	3TC, AZT, LPV/r
10	14.0	M	9.0	UD	3TC, AZT LPV/r
11	9.0	M	9.0	UD	3TC, AZT, LPV/r
12	6.2	M	6.0	UD	3TC, AZT, LPV/r
13	11.1	F	6.0	UD	3TC, AZT, LPV/r
14	16.9	M	0.5	UD	Elvitegravir/cobicistat/FTC/TDF
15	15.1	M	0.5	UD	Elvitegravir/cobicistat/FTC/TDF
16	12.2	M	3.0	12,592	3TC, AZT, NVP
17	11.5	F	11.5	29,252	3TC, AZT, LPV/r
18	14.9	F	3.0	UD	3TC, AZT, LPV/r
19	0.5	M	0.5	298,720	3TC, AZT, LPV/r
20	11.0	F	6.0	UD	3TC, AZT, DTG
21	16.3	M	1.3	896,938	3TC, AZT, DTG

ART: Antiretroviral treatment, AZT: zidovudine, DTG: dolutegravir, EFV: efavirenz, F: female, FTC: emtricitabine, HIV: human immunodeficiency virus, LPV/r: lopinovir/ritonavir, M: male, n: number of patients, NVP: Nevirapine, TDF: tenofovir disoproxil fumarate, UD: undetermined, 3TC: lamivudine.

Among HIV-monoinfected children, 1 patient (4.7%) had a history of previous splenectomy, 2 patients (9.5%) had leukopenia, 2 patients (9.5%) had anemia, and 2 patients (9.5%) had thrombocytopenia. Liver transaminases, blood urea, and creatinine levels were normal in all subjects. Two patients (9.5%) had increased LDL-cholesterol levels while triglyceride levels were increased in 3 children (14.2%). Undetectable (<45 copies/mL) viral load was found in 15 patients (71.4%). US examination revealed hepatosplenomegaly in 1 (4.7%) patient, grade 1 hepatosteatosis in 1 (4.7%) patient, and hepatosteatosis and minimal heterogeneity of the liver in 1 (4.7%) patient. Normal US findings were observed in 18 patients. 

All patients were receiving ART with a median duration of 6 (0.4–16.0) years. These drugs were lamivudine (3TC) + zidovudine (AZT) + lopinovir/ritonavir (LPV/r) (n = 13, 61.9%), AZT + LPV/r + tenofovir disoproxil fumarate (TDF) (n = 2, 9.5%), efavirenz (EFV) + 3TC + dolutegravir (DTG) (n = 1, 4.7%), elvitegravir/cobicistat + emtricitabine (FTC) + TDF (n = 2, 9.5%), 3TC + AZT+ DTG (n = 2, 9.5%), and 3TC + AZT + Nevirapine (NVP) (n = 1, 4.7%).

Stiffness (measured via kPa and m/s mode) of the liver, spleen, and both kidneys of all HIV-monoinfected children was significantly higher than that of healthy subjects (Table 3). Eighteen patients with normal US findings also had higher stiffness values compared with control subjects (P < 0.001). 

**Table 3:** Comparisons of stiffness values between patients and control group.

Parameters		Patients (n: 21),median (min–max)	Control group (n: 37),median (min–max)	P-value
Liver SWE	kPa	8.6 (4.4–25.5)	6.2 (5.1–8.1)	<0.001
	m/s	1.7 (1.2–2.9)	1.5 (1.3–1.6)	<0.001
Spleen SWE	kPa	18.7 (15.1–28.9)	16.8 (1.6–22.8)	0.002
	m/s	2.5 (2.1–3.0)	2.4 (2.0–2.7)	0.020
Right kidney SWE	kPa	18.4 (12.9–22.9)	14.1 (8.4–23.6)	<0.001
	m/s	2.4 (2.0–2.6)	2.0 (1.6–2.8)	<0.001
Left kidney SWE	kPa	18.2 (10.1–21.8)	14.4 (12.5–23.6)	0.020
	m/s	2.3 (1.8–2.6)	2.1 (2.0–2.8)	0.022

SWE: Shear wave elastography. Bolded values are statistically significant.

When the patients with detectable viral load (n = 6) and undetectable viral load (n=15) were compared, no significant difference was observed in terms of stiffness values of the liver, spleen, and both kidneys. When the stiffness values of the liver, spleen, and both kidneys of the patients with disease duration of more than 5 years and the patients with disease duration of less than 5 years were compared, no significant difference was obtained.

## 4. Discussion

According to the reported statistics, by the end of 2017, 36.9 million people, 1.8 million under the age of 15, were living with HIV [16]. Countries in sub-Saharan Africa and the Caribbean have the highest national rates of adult HIV prevalence. About 4.2% of them die during follow-up. Turkey has also been affected by the global HIV epidemic. Since the first report of 3 HIV patients in 1985, new cases have progressively increased. By the end of the 2017. 17,884 HIV-infected patients, 563 of them under 20 years of age, were living in our country [17]. This number may be underestimated due to the asymptomatic period of the disease lasting for years and inadequate admission to healthcare facilities. There is also a substantial amount of newly acquired HIV infection incidence among children in the world. Nearly 180,000 new HIV-infected children were diagnosed in 2017. For the clinicians who are dealing with HIV infection, children are particularly important and should be evaluated uniquely since they have increased life expectancy. Noninvasive radiologic methods that can predict early diagnosis and guide treatment are very helpful. Among those, SWE has been promising in recent years.

HIV and hepatitis C coinfection has an increased mortality rate compared with HIV monoinfection [18]. Coinfection increases liver damage and fibrosis. Pérez-Latorre et al. [19] reported elevated liver stiffness in coinfected patients. They also concluded that stiffness values above 12 kPa can be used to determine liver fibrosis. On the other hand, HIV monoinfection can also cause liver fibrosis because of HIV-induced immune dysregulation [20]. HIV monoinfection is an independent risk factor for liver fibrosis. Typical mechanisms of liver disease in HIV-infected patients include oxidative stress, mitochondrial injury, lipotoxicity, immune-mediated injury, cytotoxicity, toxic metabolite accumulation, vasculopathy, systemic inflammation, and nodular regenerative hyperplasia [1]. In a strain elastography study evaluating 101 HIV-monoinfected patients performed by Sulyok et al. [21], increased liver stiffness values were an indication of significant fibrosis. In our study, different from that work, we focused on monoinfected children with a newer technique called SWE. Similarly, we found increased liver stiffness values in HIV-monoinfected children compared with healthy subjects. Patients with normal US findings also had increased stiffness values. This increase may be due to HIV-related long-term inflammation and/or cytopathic effects.

The spleen is the largest lymphoid organ and the site of immune responses to blood-borne pathogens. HIV infection causes a global increase in natural regulatory T cells as well as T follicular helper cells in the spleen. Chronic HIV infection affects expression of genes implicated in stimulation, inhibition, and signal transduction [3]. In addition, architectural changes in the spleen have been reported secondary to cirrhotic or noncirrhotic portal hypertension in HIV infection [22]. HIV can cause malignancy (lymphoma) since it induces B and T cell differentiation in lymphoid organs like the spleen [23]. 

Kidney disease in HIV-infected children can vary from glomerular (such as HIV-associated nephropathy HIV immune complex kidney disease and thrombotic microangiopathy) to tubule-interstitial involvement (such as tubular injury and interstitial nephritis) and rarely vasculitis. Glomerular and tubular-interstitial involvements can cause acute and chronic renal disorders [24]. In our study, stiffness values of the spleen and kidneys were found to be significantly increased. Furthermore, this noticeable change was observed when blood parameters and radiologic findings were still normal. When the long-term cytotoxic effects of HIV on children are considered, SWE seems to be promising in the earlier detection of these changes. As far as we know, this is the first study evaluating spleen and kidney stiffness by SWE in HIV-monoinfected children. 

The patients group constituted HIV-infected children who were called to the outpatient clinic for a routine control every 3 months. Both congenital and acquired HIV-infected patients were evaluated in the present study. The youngest patient was a 6-month-old infant who also demonstrated increased stiffness in his intraabdominal organs, which may be explained by intrauterine exposure to HIV and antiretroviral agents since the beginning of the early embryonic developmental period. In addition to clinical examination, blood tests, urine analysis, and US examination were also performed regularly during the visits. Treatment, additional examinations, or follow-up decisions were based on a multidisciplinary approach for the cases with pathological findings. However, isolated SWE values are not yet included in the routine consultation algorithm in our hospital. The present preliminary study results can be used to update this algorithm. 

In the present study, possible liver fibrosis or damage of kidney and spleen parenchyma, which may be thought to cause stiffness change, could not be proven histopathologically. This is mostly because the patients were asymptomatic and lacked deterioration in biochemical tests. Therefore, we preferred to follow those patients closely in order to avoid an invasive intervention. Longer studies with pathological sampling may be the subject of future work. 

In the pediatric era, we think that routine SWE examination together with abdominal US may be very useful. Since it is a systemic infectious disorder, HIV seems to be affecting intraabdominal organs concomitantly rather than a localized involvement. In routine abdominal ultrasound examinations, increased stiffness values in solid parenchymal organs may be an indication of a systemic infectious disease such as HIV, even if the US findings are normal. In the present study, the median values for the HIV patients were 8.6 kPa and 1.7 m/s for the liver, 18.7 kPa and 2.5 m/s for the spleen, and approximately 18.3 kPa and 2.4 m/s for the kidneys. These values may vary with systemic diseases such as metabolic and collagenous disorders or other infections. Whether coinfection can change these values can be an area of further research and would add more information to our hypothesis. Since the liver transaminases, blood urea, and creatinine levels were normal in the patients of our study, we can claim that SWE may be superior to laboratory parameters in detecting early parenchymal changes without deterioration of organ function. Based on the results of the present study, we think that SWE can be used as an US assistant for noninvasive techniques in this manner. 

Detectable viral load is directly correlated with HIV progression. Moolasart et al. [25] reported increased incidence of influenza-like illnesses and opportunistic infections in patients with high viral load. Although our study cohort was limited, no significant difference was observed in terms of organ stiffness among patients with high and undetectable viral load or between patients with disease duration of more and less than 5 years. Parisi et al. [26] found no correlation between HIV viral load and liver stiffness in their study evaluating 251 patients (178 with HIV monoinfection) for a period of 12–36 months. Based on these data, one may speculate that HIV RNA replication alone does not affect organ stiffness values. However, multicenter comprehensive studies with longer follow-up periods are required for further interpretation.

There are some limitations to our study. First, the number of patients was limited. Second, blood and urine analysis could not be performed in control group children. They were included in the control group according to normal history and physical and sonographic examinations. Also, liver fibrosis and kidney or spleen injury, which are thought to cause increased stiffness, have not been proven by histopathological specimens. The other limitations were that the mean values were determined only in HIV-monoinfected patients and the effects of coinfection were not investigated. Finally, one of the operators was the practitioner and the other was the observer for SWE measurements in this study. Common consensus was reached for all reliable data, obtained when the lines were parallel and smooth in propagation mode. Intra- and interoperator reliability and repeatability were not evaluated.

In conclusion, the stiffness values of the liver, spleen, and both kidneys in HIV-monoinfected children were evaluated via US and SWE and were compared with those of healthy children. Although US findings and renal and liver function tests were within normal limits, the stiffness values of the liver, spleen, and kidneys were found to be higher than in healthy subjects. Simultaneous increase in stiffness in intraabdominal solid organs may be an indicator of a chronic systemic infectious disease such as HIV. It may be useful to perform SWE for children when US is indicated.

## References

[ref0] (2017). Mechanisms of liver disease in patients infected with HIV. BMJ Open Gastroenterology.

[ref1] (2018). C virus infection in HIV seropositive individuals & their association with risk factors: a hospital-based study. Indian Journal of Medical Research.

[ref2] (2015). HIV-infected spleens present altered follicular helper T Cell (Tfh) subsets and skewed B cell maturation. PLoS One.

[ref3] (1994). HIV and T cell expansion in splenic white pulps is accompanied by infiltration of HIV-specific cytotoxic T lymphocytes. Cell.

[ref4] (2018). HIV-associated nephropathy in Africa: pathology, clinical presentation and strategy for prevention. Journal of Clinical Medicine Research.

[ref5] (2018). Increasing prevalence and risk of chronic kidney disease in human immunodeficiency virus-infected individuals: changing demographics over a 6-year period. Journal of Infectious Diseases.

[ref6] (2018). Evaluation of the pancreatic tumors by transabdominal shear wave elastography: preliminary results of a pilot study. Medical Ultrasonography.

[ref7] (2018). The value of quantitative shear wave elastography in differentiating the cervical lymph nodes in patients with thyroid nodules. Journal of Medical Ultrasonics.

[ref8] (2018). Renal shear wave elastography for the assessment of nephron hypertrophy: a cross-sectional study in chronic kidney disease. Journal of Medical Ultrasonics.

[ref9] (2017). Testicular volume and elasticity changes in young children with undescended testes. Medical Ultrasonography.

[ref10] (2013). Breast elastography: the technical process and its applications. Diagnostic and Interventional Imaging.

[ref11] (2013). EFSUMB guidelines and recommendations on the clinical use of ultrasound elastography. Part 1: Basic principles and technology. Ultraschall in der Medizin.

[ref12] (2013). EFSUMB guidelines and recommendations on the clinical use of ultrasound elastography. Part 2: Clinical applications. Ultraschall in der Medizin.

[ref13] (2017). Diagnosis of fibrosis and activity by a combined use of strain and shear wave imaging in patients with liver disease. Digestive Diseases.

[ref14] (2017). HIV-associated complications: a systems-based approach. American Family Physician.

[ref15] (2018). The Fact Sheet HIV / AIDS.

[ref16] (2018). Hacettepe Üniversitesi HIV / AIDS Tedavi ve Araştırma Merkezi (HATAM).

[ref17] (2017). and mortality among HIV-positive individuals. AIDS.

[ref18] (2016). Prognostic value of transient elastography in human immunodeficiency virus-infected patients with chronic hepatitis C. Open Forum Infectious Diseases.

[ref19] (2012). D’Acunto K et al. Journal of Infectious Diseases.

[ref20] (2017). Hepatic fibrosis and factors associated with liver stiffness in HIV mono-infected individuals. PeerJ.

[ref21] (2012). Splenomegaly and variceal bleeding in a ten-year-old HIV-infected girl with noncirrhotic portal hypertension. Pediatric Infectious Disease Journal.

[ref22] (2017). Treatment of HIV-associated lymphomas: the latest approaches for optimizing outcomes. Oncology.

[ref23] (2018). A review of renal disease in children with HIV infection. Infectious Diseases (London, England).

[ref24] (2018). The effect of detectable HIV viral load among hiv-infected children during antiretroviral treatment: a cross-sectional study. Children.

